# The Long and Winding Road to Innovation

**DOI:** 10.5041/RMMJ.10215

**Published:** 2015-07-30

**Authors:** Rafael Beyar

**Affiliations:** Director, Rambam Health Care Campus and Professor of Medicine and Biomedical Engineering, The Rappaport Faculty of Medicine, Technion-Israel Institute of Technology, Haifa, Israel

**Keywords:** Animal experiments, early feasibility studies, innovations, inventions, medical technology, regulatory approval

## Abstract

Medicine is developing through biomedical technology and innovations. The goal of any innovation in medicine is to improve patient care. Exponential growth in technology has led to the unprecedented growth of medical technology over the last 50 years. Clinician-scientists need to understand the complexity of the innovation process, from concept to product release, when working to bring new clinical solutions to the bedside. Hence, an overview of the innovation process is provided herein. The process involves an invention designed to solve an unmet need, followed by prototype design and optimization, animal studies, pilot and pivotal studies, and regulatory approval. The post-marketing strategy relative to funding, along with analysis of cost benefit, is a critical component for the adoption of new technologies. Examples of the road to innovation are provided, based on the experience with development of the transcatheter aortic valve. Finally, ideas are presented to contribute to the further development of this worldwide trend in innovation.

## INTRODUCTION

Over the last 50 years medicine has advanced dramatically, and medical care has been completely transformed. Medicine is driven by science, technology, and innovations, which are growing exponentially. Improved patient care is the ultimate motivation for technological and scientific innovations. However, the initial and ongoing availability of these medical technologies depends on commercial success for the companies developing and eventually marketing them. Therefore, many stakeholders are involved and motivated throughout the process, including investors who many times share the motivation of improved patient care. While initial funding may be available from philanthropic sources, or government and international funds, the incentive for the funding of these ideas, both at the earliest stages and later, is a financial return for the investors. The value of new technology in medicine is far greater than its cost.^1^ There are many examples in almost any medical discipline of how technology has totally changed medical practice in the field. Over the last 50 years, the use of cardiac imaging in patients with cardiovascular diseases has led to a deeper understanding of disease pathophysiology, facilitating new therapeutic methods and percutaneous transcatheter techniques. For example, understanding coronary artery disease first led to the development of coronary bypass surgery, and later to percutaneous revascularization using balloons and stents. The technologies that enabled this transformation included novel materials, disruptive engineering, molecular and genetic methods for drug development, and combining local drug release with new medical devices. The technological background continues to change, with new metals, more precise imaging, innovative navigation methodologies, ongoing drug development, deeper understanding of genetics, the ability to scan genetic sequences at low cost with high precision, and an interconnected digital world that continuously shares and updates information worldwide.

**Figure 1:**
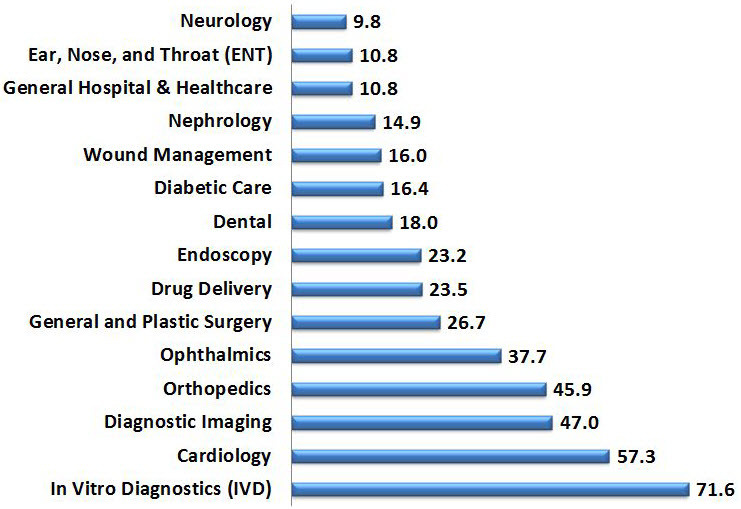
Projected Medical Technology Sales ($million) by Field Based on data from EvaluateMedTech, 2014.^2^

It is therefore no surprise that medical technology is advancing in almost every medical specialty. Worldwide projected medical technology sales by field are provided in Figure 1.^2^ Note that *in vitro* diagnostics, cardiology, imaging, orthopedics, and ophthalmology are the largest markets. Nevertheless, every field in medicine is involved in the technological revolution in health care.

When observing the various needs across medical disciplines, innovations offer new solutions for diagnostic modalities to guide therapies, for surgical and therapeutic interventions, for shifting of therapies from the clinic to home care, for assistance in routine hospital work, for helping and educating patients, for minimizing hospital stays, and for producing better therapeutic outcomes at reduced cost. The overall medical market requires strong evidence that a new technology is beneficial. This is required by both the regulatory bodies and those that decide and prioritize reimbursement at the government or institutional level, using input from the professional medical societies. Therefore, adoption of new technologies by the medical world and patients critically depends upon high-quality scientific clinical data that also have a major impact on reimbursement decisions; hence, the regulatory process becomes of utmost importance.

**Figure 2:**
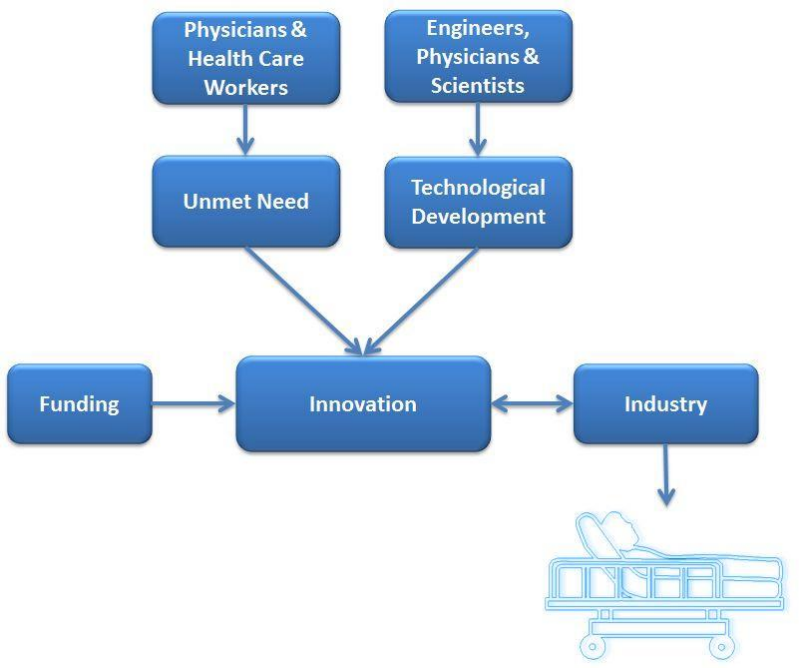
Factors that Lead to, Facilitate, and Interact with Innovations to Bring New Technology to the Patient Bed

Foundational to this development is innovation (Figure 2), which is tightly linked to therapeutic, diagnostic, or analytic unmet needs and to daily clinical practice.^3^ Medical progress comprises multiple innovations translated to contemporary clinical practice. In general, the unmet needs are highlighted by practicing physicians, while the technological and scientific solutions come from teams of engineers, physicians, and scientists. Implementation and availability of innovations for clinical use is facilitated worldwide by major life science industries. In addition, one must comply with regulations aimed at assuring the safety of the new clinical method as well as the ethics of the medical applications with regard to the patient. Life science companies today focus on medical devices, imaging, pharmacology, biotechnology, and information technology in medicine. The ability to connect electronically with any individual through smartphones and wearable digital technologies is creating a completely new environment for medical care, with a huge potential for individualized care and robust off-hospital follow-up in the future of medicine, as illustrated by Topol.^4^ This paper provides an overview of the process of innovation from concept to final product, with projections for the unique changes needed in the future.

## THE INNOVATION PROCESS

**Figure 3:**
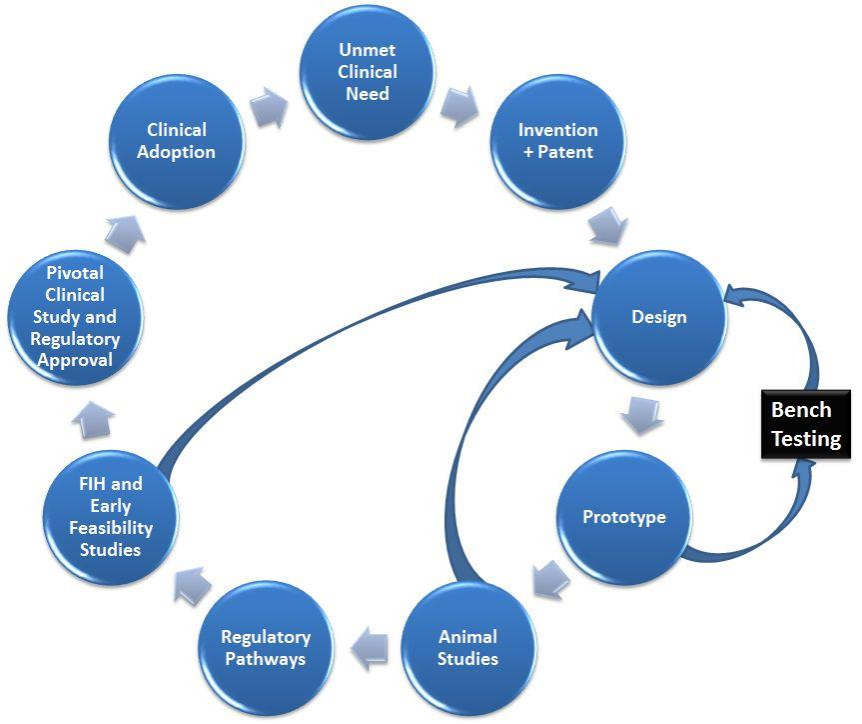
The Innovation Cycle An unmet need leads to an invention. The design and prototype go through an iterative process of optimization, followed by animal and clinical studies that are linked to the regulatory pathways. Following a successful path, regulatory approval and clinical use bring in the next unmet needs to be addressed.

The process of drug or device development begins with an invention that generates an intellectual property designed to solve an unmet need. This is followed by the innovation process^5^ outlined in Figure 3.

Foundational to every invention is a full and broad understanding of the field related to the disease to be treated or cured, the currently available solutions, and a clear delineation of the unmet need(s) in that field. A completely new technological solution is defined as a disruptive technology; an improvement or competing solution to a current technology is defined as an evolutional technology. The need must be directly associated with qualitative and quantitative market research in the relevant field. This requires surveys, interviews, and other data-mining processes to evaluate the potential market for the invention. An innovation greatly relies on the technological developments proposed by the engineer, the scientist, or the physician-scientist. The innovative process sparked by the invention depends upon multiple phases of funding and involvement of the industry to help take it through the early and later phases of commercialization.

How are ideas or inventions generated? The idea can come from individuals or may be facilitated by group brain-storming. Based on the defined need, physicians, engineers, and entrepreneurs may join a group session of brain-storming to generate several possible solutions to the need. These are then reduced to practical inventions.

The road from invention to clinical practice is long, with many steps (Figure 3). The issues of patentability, regulation, reimbursement, patient and physician acceptance, and market size are all factors in the ability of the company to sell the product for some period of time and at a price that allows ongoing availability of the product. The solution that is proposed by an invention should be clearly defined in simple terms or drawings that can be protected by appropriate patent registration. This is followed by the building of a rough prototype from simple materials, proof of concept, and introduction of changes as needed.^6^ Hence, this is an iterative process that may lead to the writing of several patents to protect the intellectual property of the inventors. In addition, virtual prototyping, using sophisticated software programs, may be used or may replace some stages throughout the process of prototype building and testing. Bench testing with the prototype is needed to show clearly the principles of the invention. Proper documentation is required for future proof of the date of the invention, should other similar ones be developed in other parts of the world within the same time frame. It should be remembered that the same triggers that spark individual inventions may potentially affect other individuals in the world to come up with similar solutions.

The invention is then rigorously tested, and questions are addressed regarding the significance of the solution. Expert views are explored and additional considerations addressed, such as the need for animal and human studies, other existing patents that could possibly block the ability to implement the innovations without the consent of the competitors, and the potential clinical market—taking into consideration current and unknown future competition.

An important factor in strategy planning that needs to be addressed as early as possible is the regulatory pathway required for clinical approval.^7^,^8^ The world’s largest regulatory body is the US Food and Drug Administration (FDA). While the FDA specifically addresses the US market, their approval has implications worldwide. Other established regulatory pathways are the CE mark regulations, and regulatory authorities in Europe, Canada, Australia, Japan, and China. The cost of the regulatory process depends on the approval track (e.g. in the USA 510(k) pathway versus a full premarket approval, PMA, track), on the number of patients needed for a pivotal clinical study, the type of study, and the required follow-up. It is not unusual for a company to invest US$150 million overall to go through the full pre-market approval process.

The early product development process is of critical importance and often determines the clinical and commercial success. Various alternatives should be considered in order to arrive at an adequate prototype product design. The design should be tested for various clinical anatomies. Accelerated wear testing is part of the requirements for approval for almost any device that is under continuous and cyclic stress patterns. The most appropriate design is then selected and “frozen” for the preclinical bench and animal phases. It is important to remember that further design modifications are likely to be required during the animal phase. Any design change will then require additional bench and animal testing. Hence, the best possible design should be achieved in order to have minimal iterations.

Animal studies are a crucial step in any device development.^6^ The goal of animal studies is to establish the biological proof-of-concept principle at an early stage of medical device development, to validate efficacy of the device in real anatomy and physiology, to test biocompatibility, and to obtain the preclinical safety data needed for submissions for early feasibility human clinical trials. The animal models should be carefully selected, to get as close as possible to the human condition. The required number of animals is defined based on previous experience with comparable devices and statistical parameters. Advanced preclinical studies that are performed under strict good laboratory practice (GLP) standards should be reserved until the device achieves a formal design freeze stage. Adverse outcomes should not be disregarded but should be solved during the animal experimental phase as they may predict clinical safety events.

Once the device has been tested in animal models, clinical studies may be required to prove its function in the human anatomy, and to provide the clinical data for safe and effective use required for device approval. If there are similar devices on the market and adequate preclinical data are provided, human studies may not be necessary.

The first-in-human (FIH) study is of crucial importance. Here the device is tested for the first time in the living human anatomy. Changes in the design are often required at this phase. It is of critical importance for the physicians to be well trained with the technology, use of bench and animal models, and that experienced investigators are involved. If the inventor is the practicing physician, he or she has an inherent conflict of interest which has to be declared and resolved. He cannot be part of the team that handles the data that are generated for proof of the safety and efficiency of the device, but at the early stages of the device-testing in patients the physician may be part of the clinical team, as his or her experience offers a greater benefit to the patient. Strict approval rules and patient informed consent apply here independent of where the study is conducted. Disclosure to the patient is of utmost importance as well as proper disclosure in presentations and publications. Later in the study, only independent investigators should be responsible for the data handling and reporting.

Randomized clinical trials (RCT) of the new device tested against current medical practice are often required for novel technologies.^9^ This is a major critical phase for device approval and is equivalent to phase III studies in drug development. The studies can be designed to show either superiority or non-inferiority with respect to current practice methods.

## IDEA PROTECTION AND PATENT FILING

Intellectual property protection has become a fundamental step in any new development whether the patent is developed within an academic or other environment.^10^ Clearly, improved patient care is the ultimate motivation for technical innovations. However, the initial and ongoing availability of these medical technologies depends on commercial success for the companies developing and eventually marketing them. The issue of patentability is critical to the ability of the company to sell the product based on the intellectual property for a defined period of time, and at a price that allows ongoing availability of the product. At an early phase of the invention, a patent search must be made to ensure that the idea has not been conceived elsewhere and a patent applied in the primary location. Patent search engines provided by Google or other sites are excellent tools for initial screening but may not provide an up-to-date picture. Hence, an expert patent office should be consulted. A provisional patent application usually gives protection for 1 year and allows the necessary time frame for a full patent application. It is important to apply for the patent before any publication, YouTube movie, or abstract presentation, as these are dated and could pose a difficulty should the patent application be challenged. It is important to solve the ownership matter early, when filing a patent. The patent will be granted if the invention is novel and non-obvious.

## THE REGULATORY PROCESS

The regulatory process differs depending on the nation. For the two major markets, the USA and Europe, the CE mark (Europe) relates to and requires safety data, whereas the FDA (USA) requires data for both safety and efficacy.^7^,^8^ The European Union (EU) handles the regulatory process via the EU Medical Device Directive (MDD); individual EU nations have regulatory authority under the MDD. While the regulation is primarily for safety, the marketplace deals with efficacy through post-marketing publications and professional opinions.

The FDA was established in 1938 and has been responsible for approving medical devices since 1976. It is part of the US Executive Department, responsible for protecting and promoting public health through the regulation and supervision of food safety, tobacco products, dietary supplements, medications, vaccines, biopharmaceuticals, blood transfusions, medical devices, electromagnetic radiation-emitting devices (ERED), veterinary products, and cosmetics.

Medical devices range from simple tongue depressors and bedpans to complex programmable pacemakers with microchip technology, implantable devices, and laser surgical devices. They can include *in vitro* diagnostic products such as general purpose lab equipment, reagents, and test kits, which may include monoclonal antibody technology. Medical devices may also emit radiation for use in medical applications such as diagnostic ultrasound products, X-ray machines, and medical lasers.

The FDA divides devices according to risk: Class I (low risk) is assigned for devices where failure will not result in death or injury to the patient or operator; Class II (moderate risk) is assigned if malfunction could result in injury or death to the patient or operator, but not of an unreasonable risk; and Class III (high risk, life sustaining) is assigned for all life-supporting or life-sustaining devices such as ventilators and life-sustaining implantable devices.

Class I devices are exempt from approval and require only registration. Class II devices require a 510(k) application path appropriate to the proposed device. Class III devices require the PMA path, with adequate clinical trials.

An exploration of recent trends in company strategies worldwide reveals that early feasibility studies of novel devices have moved to non-US sites. In addition, over the last decade novel devices are being made available outside the US before they are available in the US. A growing concern regarding the time lag in the availability of beneficial medical devices for US patients has led the FDA to reevaluate its policy, resulting in structuring a new strategy. This strategy is aimed at facilitating early feasibility studies (EFS) in the US under the investigational device exemptions (IDE) regulations, to encourage development of useful devices without jeopardizing public health and safety.^11^

Typically EFS involve a small number of subjects, and design changes are often required. With the new regulations, EFS may include several US and non-US clinical sites concurrently. Therefore a more flexible design has been suggested in which a general program is approved and the device, as well as protocol modifications, is allowed within that program.

A trend that made EFS difficult and time-consuming has also been observed in Israel over the last 20 years. Despite the fact that Israel had become a start-up nation in medical innovation^12^ with over 1,300 life science companies founded,^13^ the EFS were often performed in other countries. This is detrimental to patients and the medical profession in Israel. The patients are deprived of the chance to be exposed to novel technologies at early phases, and the physicians become late adopters of new technology, instead of leading the rest of the world. The main reasons for companies avoiding studies in Israel are the difficult and time-consuming process for obtaining study approval from the regulatory bodies. While there has been some improvement in Israel’s regulatory body, the process remains long and unfriendly for Israeli companies that wish to conduct clinical studies in Israel.

Once a product has been approved and reaches the clinical market, post-marketing studies are required to test clinical performance in a population base larger than that of the narrowly defined PMA study.^14^ Real-life experience should be adequately monitored, as this is the time for discovering new adverse events due to the new technology. The benefit for patients by gaining experience and obtaining more data is obvious. The same value should also be available for the company, and it may also guide it in its market strategy. The FDA does encourage post-marketing studies and provides guidance for the requirements for such clinical studies.^15^

## FUNDING

Innovation funding is a major challenge in itself.^16^ At very early stages the sources of funding may be competitive grants, national programs, and private investors seeking novel ideas. Later, incubator programs, strategic partners, and venture capital funds enter the game. Investors take into consideration the nature of the technology, evidence that it will present a solution to the clinical problem, and the potential markets. A clear regulatory path, a reimbursement strategy, and a strong management team are important factors. Traditional advanced funding sources are venture capital funds and major device companies. Other possible sources of funding are small business development grants (SBIR) from the US government^17^ or the recent novel crowd funding. Obviously, continuous funding is critical for the ongoing development of a product. Cessation of funding at early stages is often detrimental and referred to as the “death valley” for device development.

Song et al.^18^ published a meta-analysis on success rates of new ventures. In this empirical study of 11,259 new ventures in the USA between 1991 and 2000, only 36% survived to 3 years and 20% survived to 5 years. Most of the drop happens in the first phases of screening, business analysis, and development. The funding environment has changed today as the cost of medical technology is higher. Yet, medium and large companies depend upon academics and entrepreneurs to fill the pipeline of innovative products, and the need of companies for a strong medical technology pipeline is great.

## MARKET APPLICATION AND REIMBURSEMENT

Naturally, after a new technology is approved it must enter the appropriate market and compete against existing technologies. Therefore, reimbursement strategies, complementary to strong clinical evidence generated by trials, are of critical importance to the success of a device.^19^ There are many reimbursement mechanisms in different parts of the world with huge variability among them. In general, reimbursement for a new device requires evidence to support claims that the new technology leads to better patient outcomes at lower cost than existing solutions.

In Israel, once a new technology is approved for use, a mechanism exists for budgeting introduction of that new technology to the general public. A public committee for the expansion of the “basket” of health services was formed at the beginning of the century by the Ministry of Health and Finance in order to decide about funding of new drugs and technologies on a yearly basis.^20^ This public committee is composed of medical experts, medical ethics and economy experts, representatives of the health insurance companies, public health experts, and public representatives. The role of the committee is to advise the Minister of Health regarding which technologies and drugs should be approved for public funding within the allocated budget. Over the last several years, 400–500 applications have been presented to the committee annually, out of which 80–100 technologies are approved. The criteria for prioritization include professional evidence for safety and efficiency, epidemiological assessment of the number of patients and the needs, assessment of the current solutions to the presented needs, and evaluation of the evidence that exists in the medical literature. Social and legal parameters are also applied to the decision-making process. Saving lives, prolonging life, and improving quality of life are all taken into consideration. Once the basket expansion is approved the new technologies become available to the entire public through the public insurance bodies.

By this mechanism, new drugs are added to the portfolio every year. The approved technologies, ranging from pharmaceuticals and medical devices to technologies with proven benefit to the public, treat the full gamut of medical conditions. The majority of funding is channeled through the public health insurance companies. While this is an excellent mechanism to provide expansion of drugs and technologies, it does not provide adequate technological advancement to hospital-based technologies. A specific mechanism for funding increasing hospital costs due to new technologies, accounting for patient quality of care, cost effectiveness, and possible cost saving should be considered.

## EXAMPLE OF DISRUPTIVE NEW TECHNOLOGY: TRANSCATHETER AORTIC VALVE REPLACEMENT

Surgical replacement of the aortic valve is the current standard of care in severe aortic stenosis. Table 1 lists the events that led to development of the transcatheter aortic valve replacement (TAVR). The need for transcatheter treatment led to aortic balloon valvuloplasty, introduced by Cribier in 1985.^21^ However, this therapy was associated with a high complication rate and early recurrence of stenosis. Andersen was the first to develop an artificial valve suitable for percutaneous implantation and showed feasibility in a swine model. In 1989, the first pig survived the implantation. Yet, despite the evidence, nobody believed in the technology. Andersen had severe difficulties in publishing until his paper was finally published in the European Heart Journal in 1992.^22^ A patent by Andersen et al.,^23^ granted in 1995, with a priority date of 1990, described a valve prosthesis for implantation in the body and a “catheter for implanting such valve prosthesis.”

**Table 1:** Unmet Need: Percutaneous, Minimally Invasive Treatment for Aortic Stenosis–Time Frame of Development of the First Disruptive Device.

Year	Event	Reference
1985	Aortic balloon angioplasty by Alain Cribier	Cribier et al.^21^
1989	First successful experiment in pigs with porcine aortic valve in an expandable metal cage; published in 1992	Andersen et al.^22^
1990	Priority date for the Andersen patent granted in 1995	Andersen et al.^23^
1999	PVT founded and acquired rights of the Anderson patent	Datafox^24^
2002	First human implantation by Alain Cribier	Cribier et al.^25^
2004	PVT acquired by Edwards	Bloomberg Business^26^
2005	Edwards Feasibility trial launched	Barbash and Waksman^27^
2006	Initiating the pivotal FDA study–PARTNERS	Barbash and Waksman^27^
2007	CE approval granted in Europe for the Sapien^™^ valve	Barbash and Waksman^27^
2008	The Sapien^™^ valve approved in Israel	Segev and Goita^28^
2010	The Sapien^™^ valve granted public funding for non-operable surgical patients	The Prime Minister’s Office^29^
2011	FDA approval for non-operable patients	Barbash and Waksman^27^

The patent was sold for $10,000 to a small private company founded in 1999, Percutaneous Valve Technologies, Inc. (PVT), based in New Jersey, USA, with a subsidiary in Israel.^24^ After using a sheep animal model to test the safety and feasibility of the valve, the first human TAVR procedure was performed on April 16, 2002, by Cribier et al.^25^ in a 57-year-old inoperable patient with severe symptomatic aortic stenosis; the patient’s condition deteriorated after balloon aortic valvuloplasty. The TAVR procedure was successful, and the patient survived for another 4 months, subsequently dying from non-cardiac causes. The EFS registry trials launched in 2006 enrolled a total of 55 patients. The procedural success in these high-risk patients was 75%, with a complication rate of 22%.

The major pivotal trial, the PARTNER trial, was launched in 2006, with CE approval granted in 2007 and FDA approval in 2011. The randomized studies that continued showed clear benefit and prolonged life in high-risk patients, as compared to medical therapy.^30^,^31^

The Israel story is also of interest. While the engineering was done in Israel the early feasibility trials were performed in Europe, the USA, and Canada. The device was granted approval in 2008 following CE approval and was approved for public funding in 2010. The lessons to learn are that strict regulatory barriers may have led the Israeli-linked company to avoid Israel as a clinical field.

This clearly shows that introduction of a disruptive new technology depends on vision, persistence, and technical, clinical, and financial abilities to move such innovations forward. While both the patented idea^22^ and initial feasibility was demonstrated in an animal model via an academic set-up by Andersen et al.,^21^ the rest of the innovation process was carried out by the industry and went through very rough times, including a high complication rate and criticism. Later, Andersen responded: “The task was too big for us, and nobody else in Denmark could handle it. We tried, but it was impossible. The only thing that I regret a bit is that I did not contribute to developing the idea until it could be used in humans. I would have liked to have been part of that.”^32^

As shown in this story, major innovations require true collaborative efforts between individual physicians, academia, and industry, and depend upon vision and persistence that often extend well beyond the first investigator. In addition, it is clearly seen that the penetration rate of devices is significantly delayed in the USA compared with Europe (4 years later in the TAVR case). This is mostly due to the regulatory agencies in the USA which require very rigorous clinical testing of a device prior to its approval.

## PRACTICAL STEPS TOWARDS BUILDING A BIOMEDICAL-FRIENDLY INNOVATION SOCIETY

Advancing medicine through technology for improved patient care is an important value that should be adopted at all levels of education and practice. Several lessons can be learned at the institutional, the national, and the global scales. Ten recommendations to facilitate innovations are offered in Table 2. Firstly, innovation education in the medical field should be part of the curricula of medical schools, as well as engineering schools. Universities, medical institutions, and countries should develop programs that promote and support both basic and applied research; these programs should be directed towards young clinical scientists who are experiencing current medical practice with modern and fresh eyes. This should be a national priority with funding secured via legislative mechanisms since it is otherwise very difficult to compete against the high-priority needs of society. Early-stage funding for appropriate innovative ideas should be made available to physicians, engineers, and scientists. Such funding may come from government support systems such as the incubators, or other private or international sources. There is always the argument that excessive funding at an early stage with loose evaluation processes leads to a waste of efforts and resources and higher failure rates; while the other side of the spectrum is lean funding and inability to support excellent ideas. Large medical device companies should be provided with strong incentives to invest in innovations as their contribution to society.

**Table 2:** Leading Actions that Should Be Implemented to Promote Health Care Innovations.

	Task	Responsibility
1	Include medical innovations education in medical and engineering schools	Universities/academic institutions
2	Support basic and applied medical research in hospitals and universities	Governments, universities/academic institutions, foundations
3	Secure national funding for applied research through legislative mechanisms	Governments, offices responsible for medical and industrial research
4	Secure early-stage innovation funding for clinical scientists and engineers in the public and private sectors	Governments, universities/academic institutions, medical institutions
5	Promote investment in innovations by the leading medical technology and pharmaceutical industries	Voluntary through industrial consortium
6	Limit time to approval of early human feasibility studies to 3 months	Government
7	Simplify the regulatory process and make it less bureaucratic; allow consultation at early phases	Government
8	Build and promote medical innovation centers near academic medical centers	Government, industry
9	Promote collaborations between hospitals, academia, and industry through consortia and international programs	National and international initiatives
10	Develop mechanisms for funding and implementing new innovative technologies	Governments, health care providers

The regulatory process should protect the public from unproven technology, but at the same time it should be made friendlier for the investigators who seek the easiest pathway to approval. Again, it is a delicate balance between protecting the public from harm that may be caused by an inappropriate technology, and providing a more accessible and less costly mechanism for approval of new devices. A consultation mechanism between the regulatory body and the companies applying for approval should be implemented at early phases of development. It is important to remember that being over-cautious can also harm society by preventing or delaying access to much-needed new technologies. The potential damage of delaying access to successful technologies should be balanced with the damage incurred from a premature approval. To avoid delays, the regulatory pathway for human clinical studies should have a strict time-to-approval of up to 3 months from the initial applications.

To facilitate excellence in clinical innovation and promote collaboration, medical innovation centers such as biotechnological incubators should be built in proximity to academic medical centers and hospitals. The medical technology industries need to be provided with long-term support throughout innovative product development. It should be understood that innovative product development can take 5–10 years or more, and financial support throughout this journey is critical.

Every effort should be made to promote collaborations between academic hospitals and medical industry, such as through the sharing of intellectual properties and generating mutually beneficial relationships. The task should be to break down the walls that limit interactions, rather than building them. Mechanisms should be implemented to resolve conflicts of interest that may exist at the individual or institutional level. A collaborative network of hospitals, universities, and industry must develop tools to deal with such conflict appropriately.

While the principles mentioned above are global, they are particularly applicable to Israel, a nation that has led the world as a medical “Start-up Nation”^12^ and has the potential to continue to do so. Finally, implementing mechanisms for the gradual introduction of new technologies to clinical practice is of critical importance. Here the mechanism implemented in Israel via expansion of the health service basket can be a guide to the rest of the world.

## SUMMARY

The pathway for developing and introducing a new technology to the medical field for improved patient care is complex. A new invention, which must be patented to protect the intellectual property, is at the basis of every innovation process that aims to solve an unmet clinical need. The innovation process involves tedious repetitive cycles of design optimization, animal testing, and patient testing according to the rigorous criteria of regulatory bodies. Different regulatory pathways exist in different parts of the world and between different types of device classes, defined by the risk to the patient. Clinical adoption is the very last step of the innovation and is markedly affected by the benefit proven in the clinical trials, by the additional value it brings in, by its cost, and by reimbursement strategies. The future for medical device innovation is bright due to the explosive growth of technology and the dynamic features of modern medicine, but it will be limited by economic, regulatory, and ethical limitations.

## Abbreviations

EFS: early feasibility studies
EU: European Union
FDA: Food and Drug Administration
FIH: first-in-human
IDE: investigational device exemptions
MDD: Medical Device Directive
PMA: premarket approval
RCT: randomized clinical trials
TAVR: transcatheter aortic valve replacement.
